# A Novel Simulation Framework for Evaluating and Optimizing Oncology Outreach Policies in a Regional Cancer System

**DOI:** 10.1002/cam4.71771

**Published:** 2026-04-05

**Authors:** Bruno T. Scodari, A. James O'Malley, Nirav S. Kapadia, Gabriel A. Brooks, Ashlee A. Korsberg, Erika L. Moen

**Affiliations:** ^1^ Department of Biomedical Data Science Geisel School of Medicine at Dartmouth Lebanon NH USA; ^2^ The Dartmouth Institute for Health Policy and Clinical Practice Geisel School of Medicine at Dartmouth Lebanon NH USA; ^3^ Dartmouth Cancer Center Geisel School of Medicine at Dartmouth Lebanon NH USA; ^4^ Department of Radiation Oncology and Applied Sciences Geisel School of Medicine at Dartmouth Lebanon NH USA; ^5^ Department of Medicine Geisel School of Medicine at Dartmouth Lebanon NH USA

## Abstract

**Background:**

Oncology outreach is a common strategy for addressing cancer workforce shortages, where traveling oncologists commute across healthcare settings to extend specialized care. However, methods for evaluating and optimizing outreach strategies are limited.

**Methods:**

We developed a simulation framework for evaluating and optimizing potential outreach strategies and applied it to two test cases within Dartmouth Health's hub‐and‐spoke cancer system. Our approach involved sampling incident patients and visit capacity across oncologist/site pairs, assigning patients to oncologist/site pairs, and estimating the total patient travel time incurred across two groups: (1) where a portion of patient visits is shifted from the hub facility to a satellite, and (2) where the original distribution of patient visits is retained. After 5000 simulations, the mean difference in patient travel time among groups was computed to estimate patient travel time savings. We identified the optimal number of new patient visits to reallocate from the hub to satellite that maximized patient travel time savings.

**Results:**

We identified satellites in St. Johnsbury, VT and Manchester, NH as candidates for increased medical and radiation oncology outreach, respectively. At St. Johnsbury, we found that reallocating 45% of new medical oncology visits resulted in 140 (95% CI: 97–174) total hours saved. For patients whose care was shifted, this corresponded to an average of 1.07 (95% CI: 0.75–1.34) hours saved per visit. At Manchester, we found that reallocating 50% of new radiation oncology visits resulted in 55 (95% CI: 34–71) total hours saved. For patients whose care was shifted, this corresponded to an average of 1.13 (95% CI: 0.70–1.44) hours saved per visit.

**Conclusion:**

Simulations offer a valuable means to assess the effectiveness and uncertainty of potential outreach strategies. Such approaches are well positioned to provide administrative decision support for health systems and can be leveraged to inform directional investments in outreach policies.

## 1 Introduction

Oncology outreach is a common healthcare strategy for addressing cancer workforce shortages [1]. Under the outreach model, traveling medical and radiation oncologists (herein referred to as “oncologists”) commute to satellite clinics to extend specialized care on a set cadence (e.g., monthly) [2, 3, 4]. A hallmark of oncology outreach is “meeting patients where they are,” with several studies linking oncology outreach to reduced travel burden for patients [5, 6]. Outreach arrangements are particularly beneficial for rural populations, with prior research showing that Medicare beneficiaries realized one‐way travel time savings of 16 and 12 min to medical and radiation oncology outreach visits, respectively [4]. Beyond reducing geographic barriers to care, oncology outreach is also associated with improving the timeliness and utilization of cancer care [7, 8]. Given these known benefits, an important next step is advancing how health systems decide where and how to staff satellite clinics for cancer care delivery.

Under simplistic assumptions, health systems make outpatient staffing decisions using traditional physician supply and patient demand measures [9, 10]. In oncology, administrative leaders may use demand projections and attempt to balance specialist coverage accordingly, which is often reflected in oncologist contracts. However, variation in patient preferences and needs for cancer care can contribute substantial challenges in the allocation of an already constrained workforce [11]. In addition, strategies that increase physician resources at satellite clinics can be costly to implement [12], lead to physician burnout and attrition [13, 14, 15], and disrupt care coordination at the system level, making them inherently risky [16]. To better inform outreach decisions, simulations can be used to interrogate “what‐if scenarios” for hypothetical policies and quantify their associated uncertainty. Such approaches offer the ability to understand the implications of a policy with a certain level of confidence prior to implementation.

In this study, we introduce a novel simulation framework for evaluating and optimizing potential outreach policies. The crux of our approach involves sampling incident patients and visit capacity across oncologist/site pairs under two scenarios: a policy group, where a prespecified number of new patient visits is shifted from the hub facility to a satellite, and a counterfactual control group, where the original distribution of new patient visits is retained across sites. For both groups, patient‐oncologist assignments are determined using a novel algorithm that (1) accounts for the order of incident patients and their preferences, and (2) provides a computationally tractable/efficient runtime—key differentiators from previous methods. With these patient‐oncologist assignments, the total incurred patient travel time is computed for each group and stored before continuing to the next iteration. After repeating this process 5000 times, the mean difference in patient travel time between groups is calculated to estimate patient travel time savings attributable to the policy. By iteratively adjusting the amount of oncologist effort to be shifted, the simulator identifies the optimal strategy that maximizes patient travel time savings. While we recognize our approach optimizes for one element of care (patient access) and largely ignores other factors like oncologist preferences, we emphasize the utility of our methodology for thinking directionally about potential outreach investments as opposed to providing absolute clinical recommendations.

## 2 Methods

### 2.1 Data Sources

We used de‐identified data from Dartmouth Health's electronic health record (EHR) and cancer incidence data from the Dartmouth Cancer Center's Catchment Query Portal (CQP). The Dartmouth Health institutional review board approved all study protocols and issued a waiver for obtaining informed consent from human subjects prior to study commencement.

#### 2.1.1 EHR Data

The EHR included encounter‐level data for a cohort of patients with breast (female only), colorectal, and lung cancers from 2020 to 2022. Patients were originally identified using Dartmouth's tumor registry, restricted to patients older than 18 years and younger than 99 years at time of cancer diagnosis, and linked to the EHR to obtain clinical encounters and treating oncologists from 3 months prior to 12 months following diagnosis [17]. Clinical encounters were filtered for in‐person visits by excluding encounter descriptions with “letter,” “notes/messages,” “orders,” and “telehealth” references. Unique patient identifiers (e.g., residential location) were removed to de‐identify the data.

#### 2.1.2 Catchment Query Portal (CQP) Data

The CQP included counts of incident cases in the catchment region by zip code tabulation area (ZCTA) and cancer type from 2014 to 2023. We required this as a secondary data source because patient residence was unavailable in the de‐identified EHR data.

### 2.2 Generating Distributions

Using the EHR and CQP data, we estimated two distributions to motivate our test cases and use as sampling inputs for our simulations.

#### 2.2.1 Joint Distribution for Patient ZCTA, Cancer Type, and Cancer Stage

We estimated the joint distribution between patient ZCTA, cancer type, and cancer stage (herein referred to as the “patient generator”). The CQP contained information for patient ZCTA and cancer type while the EHR contained information for patient cancer type and cancer stage. We assumed that ZCTA and cancer stage were conditionally independent given cancer type, which allowed us to leverage the available data from the CQP and EHR to estimate the joint distribution for all three patient variables.

#### 2.2.2 Average Annual Patient Volume for Each Oncologist/Site Pair

Using the EHR data, we computed the average annual patient volume for each oncologist/site pair. Patient volume was defined as the number of unique study cohort patients an oncologist provided care to over a year.

### 2.3 Identifying Satellites for Increased Outreach

**FIGURE 1:**
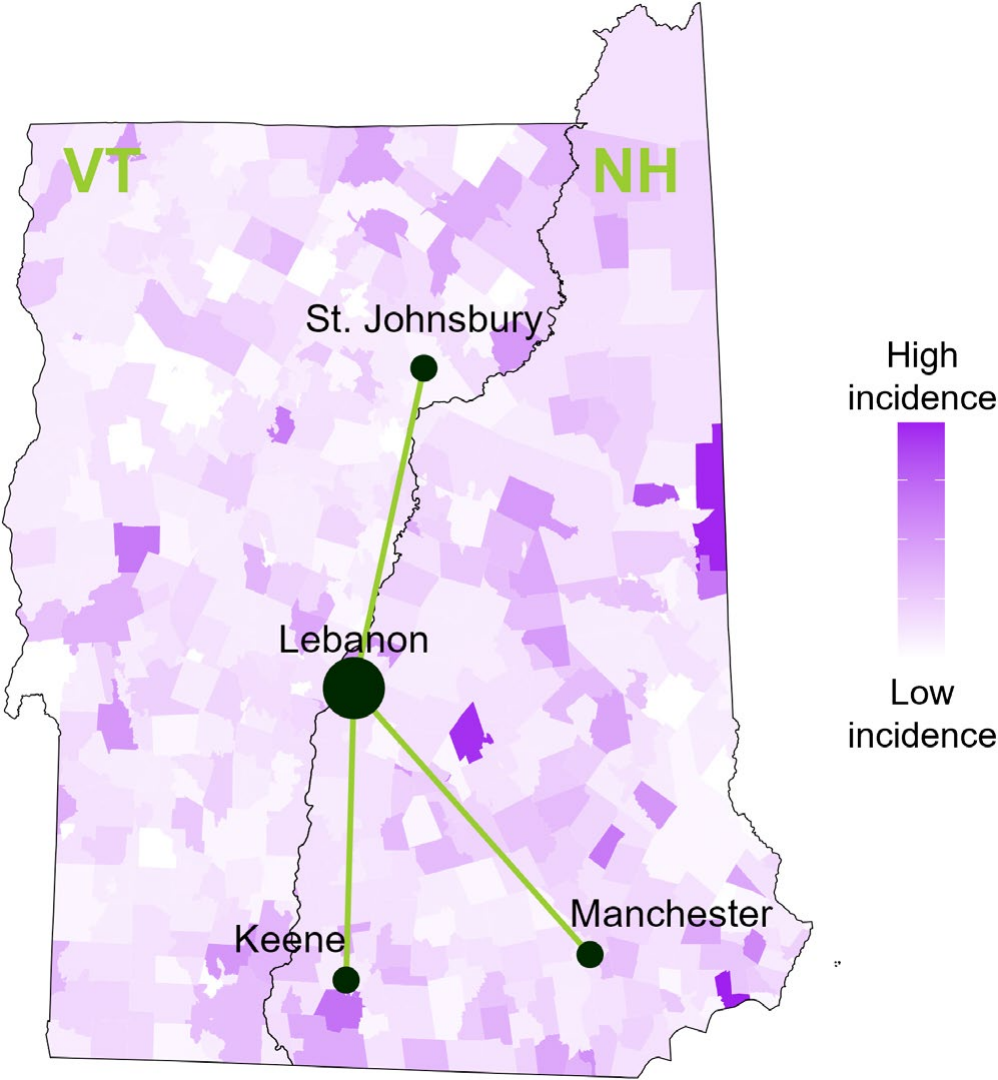
Visualization of the Dartmouth Health hub‐and‐spoke cancer system with superimposed incidence for patients with breast, colorectal, and lung cancers.

There are five major cancer clinics within Dartmouth Health's catchment area. Dartmouth Hitchcock Medical Center in Lebanon, NH serves as the “hub” hospital. The four regional satellites are in Keene, Manchester, and Nashua, NH, and in St. Johnsbury, VT. We did not consider Nashua as a candidate for increased outreach due to its geographic proximity to the larger Manchester site. We visualized the geographic dispersion of these sites with superimposed cancer incidence by ZCTA (Figure 1).

We identified candidates for increased medical and radiation oncology outreach based on empirical supply and demand metrics. Within a bootstrapped framework, we sampled the average annual number of incident cases from the patient generator; determined patient demand for medical and radiation oncology by drawing Bernoulli random variables from distributions parameterized based on each patient's respective cancer type and stage (Table S1); calculated the closest site for each patient using Hu et al.'s travel time matrix [4, 18]; and tallied the results by site. Upon repeating this process 5000 times, we estimated the number of closest patients to each site by oncology specialty. We divided these estimates by the average annual patient volume at each site to obtain a ratio of demand to supply. Satellites with ratios substantially exceeding that of the hub's (i.e., that had greater demand in relation to supply compared with the hub facility) were considered as candidates for increased outreach.

### 2.4 Simulating Oncology Outreach Policies

**FIGURE 2:**
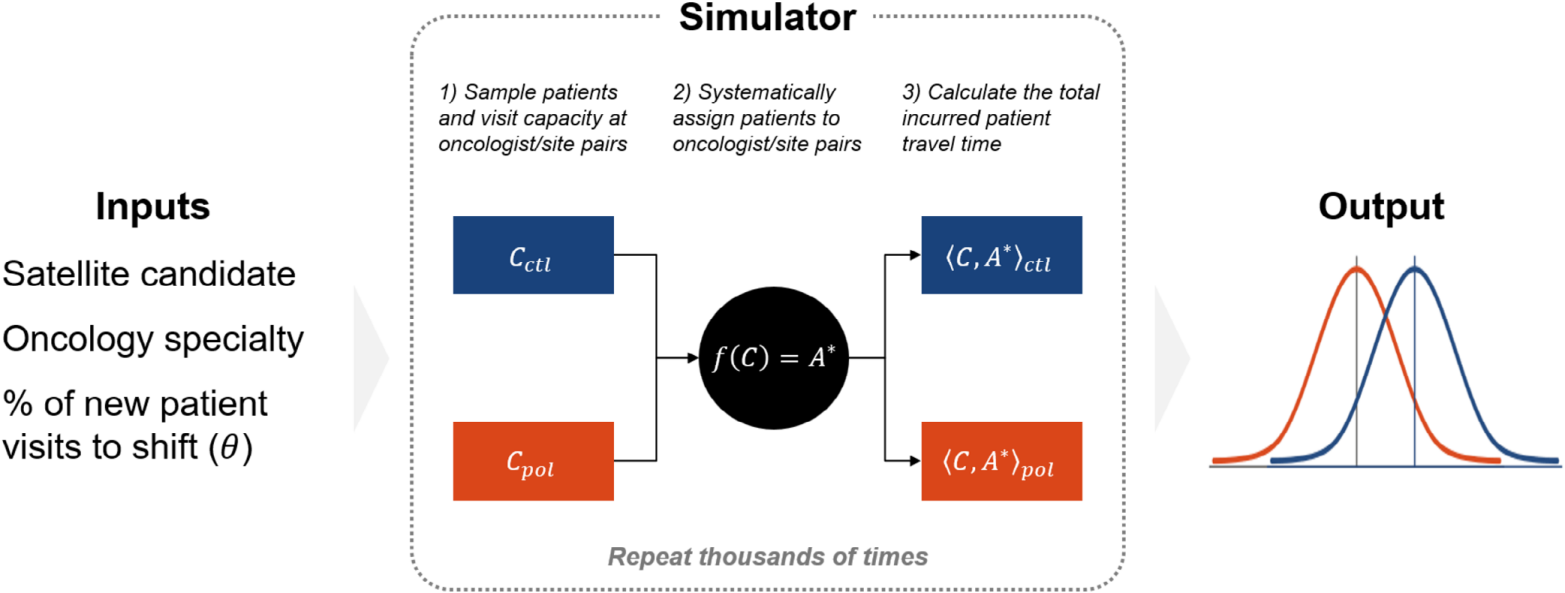
Simulation framework. To evaluate a given oncology outreach policy, a specified satellite, oncology specialty, and the proportion of new patient visits to shift from the hub to satellite (θ) are required as inputs. Based on this information, counterfactual control and policy groups are simulated by generating two separate cost matrices, solving for the assignment matrices, and calculating total patient travel times. This process is simulated 5000 times to generate outcome distributions for each counterfactual group. These distributions are compared to derive savings estimates and measures of uncertainty.

The goal of our simulation framework is to estimate the round‐trip patient travel time savings that could be captured the following year if an outreach policy is implemented. An outreach policy has three components: the satellite candidate of interest, the oncology specialty under consideration, and the proportion of annual new patient visits, θ, to reallocate from the hub hospital (to the satellite). These components are prespecified as inputs to the simulator (e.g., St. Johnsbury, medical oncology, 5% reallocation), which samples incident patients and visit capacity across oncologist/site pairs under two counterfactual groups (policy vs. control), assigns patients to oncologist/site pairs using a novel assignment algorithm, and calculates the total incurred patient travel time. Repeating this process thousands of times results in two distributions of incurred patient travel time that are compared via statistical methods (Figure 2).

#### 2.4.1 Inputs

As previously mentioned, our simulation framework requires a satellite candidate, oncology specialty, and proportion of new patient visits, θ, to reallocate from the hub to satellite as inputs. For each satellite‐specialty combination, all θ values ranging from 0% to 100% were evaluated in 5% increments.

#### 2.4.2 Simulator

Given a set of inputs, the following steps are performed in one simulation iteration. We provide a high‐level conceptual overview of these steps below and include the technical details in the Supporting Information.

(1) Sample patients and visit capacity at oncologist/site pairs.

Under a policy and control group, the simulator samples incident patients and visit capacity across oncologist/site pairs. This information is stored in two corresponding cost matrices, $C_{\mathrm{pol}}$ and $C_{\mathrm{ctl}}$, where the rows represent patients, the columns represent oncologist/site pairs with a fixed number of visits, and the elements contain the round‐trip travel time that patients would incur if they were assigned to a specific oncologist/site pair [18]. Importantly, both cost matrices contain the same patient information (in the rows) but different allocations of visits across oncologist/site pairs (in the columns).

**FIGURE 3:**
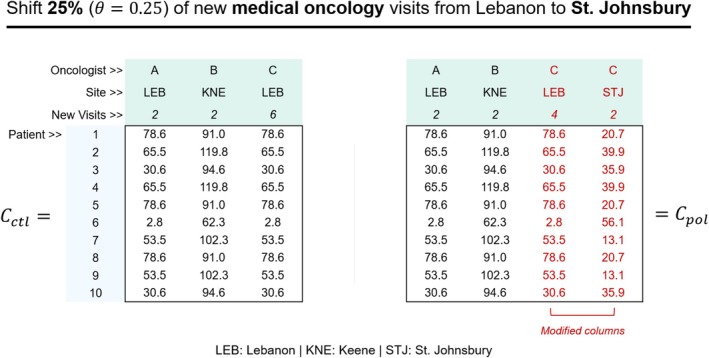
Generation of counterfactual cost matrices. Given an example outreach policy that shifts 25% of new patient visits from Lebanon to St. Johnsbury, two counterfactual cost matrices (control and policy) are generated in each simulation iteration. The row space of each matrix contains the same sample of incident cancer patients. The column space of these matrices contains different information pertinent to the distribution of new patient visits across oncologist/site pairs. In the control group, the original distribution of new patient visits at oncologist/site pairs is retained. In the policy group, 25% of new patient visits are randomly reallocated from Lebanon to St. Johnsbury, which increases its dimensionality. The elements in each cost matrix are populated with all pairwise travel times between patients and oncologist/site pairs.

As a contrived example of how these matrices are constructed, assume there are 10 patients who require medical oncology care and 3 medical oncologists who can see these patients (Figure 3). Imagine we are interested in simulating a policy where 25% (θ=0.25) of new medical oncology visits are reallocated from Lebanon to St. Johnsbury. In the control group, the historical average annual patient volume observed at each oncologist/site pair determines the number of visits allocated to each oncologist site/pair. However, in the policy group, 25% of the Lebanon‐based visits (i.e., 2 out of 8 visits) are reallocated to St. Johnsbury, resulting in one oncologist needing to apportion their time across two sites.

(2) Systematically assign patients to oncologist/site pairs.

After constructing the cost matrices, the simulator seeks to assign patients to oncologist/site pairs and store this information in assignment matrices, $A_{\mathrm{pol}}$ and $A_{\mathrm{ctl}}$. The assignment matrices contain the same row and column information as their corresponding cost matrices; however, they contain Boolean elements that indicate patient assignments to oncologist/site pairs. To solve for the optimal assignment matrix $A^{*}$ given C, we assume that patients always select the oncologist/site pair that minimizes their travel time with respect to the following constraints:

- Patients may only select one oncologist/site pair.
- Patients may only select an oncologist/site pair with available capacity.
- Patients select oncologist/site pairs on a first‐come, first‐served basis.

**FIGURE 4:**
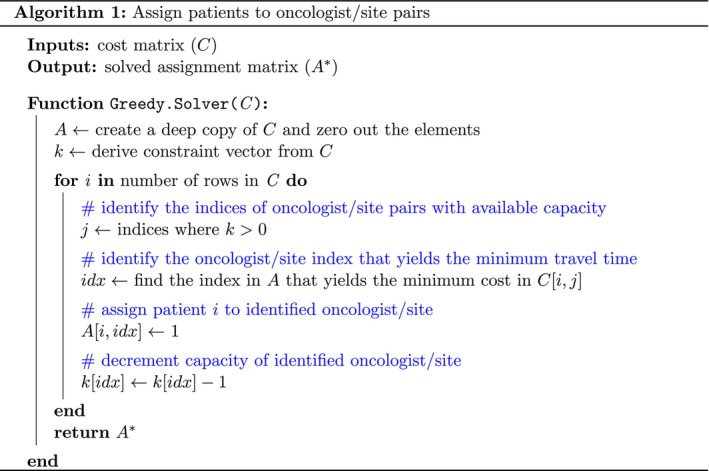
Greedy algorithm for assigning patients to oncologist/site pairs.

We implemented the above logic using a novel algorithm that assigns patients to oncologist/site pairs in a greedy fashion (Figure 4). A greedy approach was chosen to honor the order of incident patients and preserve computational efficiency.

(3) Calculate the total incurred patient travel time

After solving for the assignment matrices, the simulator calculates the total round‐trip patient travel time incurred within each group. To do so, the inner product (sum product) between each group's cost and solved assignment matrix, $\left\langle C,A^{*}\right\rangle$, is computed.

#### 2.4.3 Output

The above process is repeated 5000 times, resulting in a distribution of incurred patient travel times for each group. The patient travel time savings attributable to the policy is estimated by computing the mean difference between the policy and control groups, along with a 95% confidence interval (CI) using the percentile method.

### 2.5 Identifying the Optimal θ

Recall that we iteratively evaluated θ values from 0% to 100% (in 5% increments) for a given policy. Therefore, to identify the optimal proportion of new visits to reallocate from the hub to satellite, we plotted the estimates for patient travel time savings and 95% CIs against θ to visualize the trajectory of savings as a function of increased outreach.

### 2.6 Net Travel Time Savings

As a secondary outcome, we computed the net travel time savings after accounting for oncologist travel between sites by subtracting the incurred oncologist travel time from patient travel time savings. When calculating oncologist travel, we assumed that oncologists saw an average of 4 patients with breast, colorectal, or lung cancer per day when practicing at a satellite. As these cancers represent ≈36% of incident cases [19], this corresponds to a total of ≈10 patients per day. We performed sensitivity analysis to flex this assumption.

### 2.7 Accounting for Follow‐Up Visits

Furthermore, we calculated patient and net travel time savings for the case where all follow‐up visits occurred at the satellite. In our data, the average number of patient visits for medical and radiation oncology services was 12 and 9, respectively, which were used as multiples to calculate the total savings when accounting for follow‐up visits. We assumed that each satellite possessed the capital/infrastructure (e.g., equipment) required to support the redistribution of oncologist effort and manage follow‐up visits across all levels of case severity.

## 3 Results

### 3.1 Candidates for Increased Oncology Outreach

We identified St. Johnsbury and Manchester as candidates for increased medical and radiation oncology outreach, respectively, based on their shortages of supply relative to Lebanon (Table 1). In our simulations, we explored reallocating between 15 and 287 new medical oncology patient visits from Lebanon to St. Johnsbury and between 5 and 97 new radiation oncology patient visits from Lebanon to Manchester (Table S2). As outlined by the data, there was no radiation oncology presence in Manchester from 2020 to 2022.

**TABLE 1:** Supply & demand estimates for medical and radiation oncology services by site.

	Lebanon (hub)	Keene	Manchester	St. Johnsbury
Medical Oncology				
Demand: Proximal patient count (SD)^a^	183 (12)	96 (9)	72 (8)	175 (12)
Supply: Patient volume	297	95	105	56
Ratio	0.6×	1.0×	0.7×	3.1×
Radiation Oncology				
Demand: Proximal patient count (SD)^a^	89 (9)	47 (7)	37 (6)	85 (9)
Supply: Patient volume	97	111	0	59
Ratio	0.9×	0.4×	undefined^b^	1.4×

^a^ Within a bootstrapped framework, we sampled the average annual number of incident cases from the patient generator; determined patient demand for the given oncology specialty by drawing Bernoulli random variables from distributions parameterized based on each patient's respective cancer type and stage (Table S1); calculated the closest site for each patient; and tallied the results by site. Upon repeating this process 5000 times, we estimated the number of closest patients to each site.

^b^ Due to zero division.

### 3.2 Patient Travel Time Savings as a Function of Increased Outreach

**FIGURE 5:**
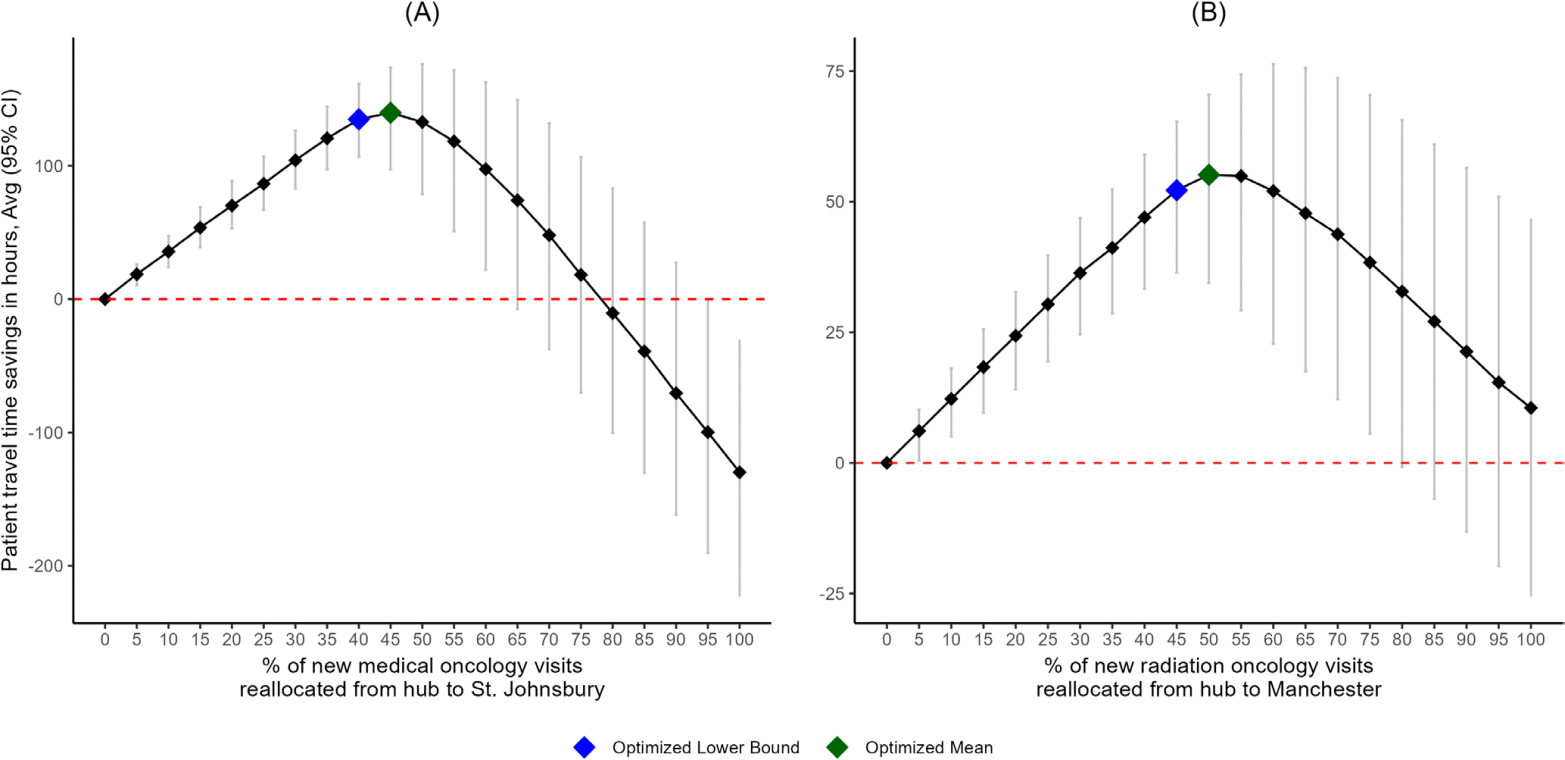
Patient travel time savings as a function of oncology outreach. (A) At St. Johnsbury, patient savings were maximized by shifting 45% of new medical oncology visits, which resulted in 140 (95% CI: 97–174) total hours saved. For patients whose care was shifted to St. Johnsbury, this corresponded to 1.07 (95% CI: 0.75–1.34). (B) At Manchester, patient savings were maximized by shifting 50% of new radiation oncology visits, which resulted in 55 (95% CI: 34–71) total hours saved. For patients whose care was shifted to Manchester, this corresponded to 1.13 (95% CI: 0.70–1.44) hours saved per visit.

For St. Johnsbury, round‐trip patient travel time savings significantly increased when shifting 5% to 60% of new medical oncology visits from the hub to satellite. Reallocating 65% to 90% of new visits resulted in no statistically significant patient savings, while reallocating ≥ 95% was deleterious (Figure 5A). Patient savings were maximized when reallocating 45% of new visits, resulting in 140 (95% CI: 97–174) total hours saved. For patients whose care was moved from the hub to the satellite, this corresponded to an average of 1.07 (95% CI: 0.75–1.34) hours saved per visit. When assuming follow‐up visits also occurred at the satellite, patient savings increased to 1677 (95% CI: 1165–2083) total hours (Table S2 & S3).

For Manchester, round‐trip patient travel time savings significantly increased when shifting 5% to 75% of new radiation oncology patient visits from the hub to satellite. Reallocating ≥ 80% of new visits resulted in no statistically significant savings (Figure 5B). Patient savings were maximized when reallocating 50% of new visits, resulting in 55 (95% CI: 34–71) total hours saved. For patients whose care was moved from the hub to the satellite, this corresponded to an average of 1.13 (95% CI: 0.70–1.44) hours saved per visit. When assuming follow‐up visits also occurred at the satellite, patient savings increased to 496 (95% CI: 310–635) total hours (Table S2 & S4).

### 3.3 Net Travel Time Savings as a Function of Increased Outreach

**FIGURE 6:**
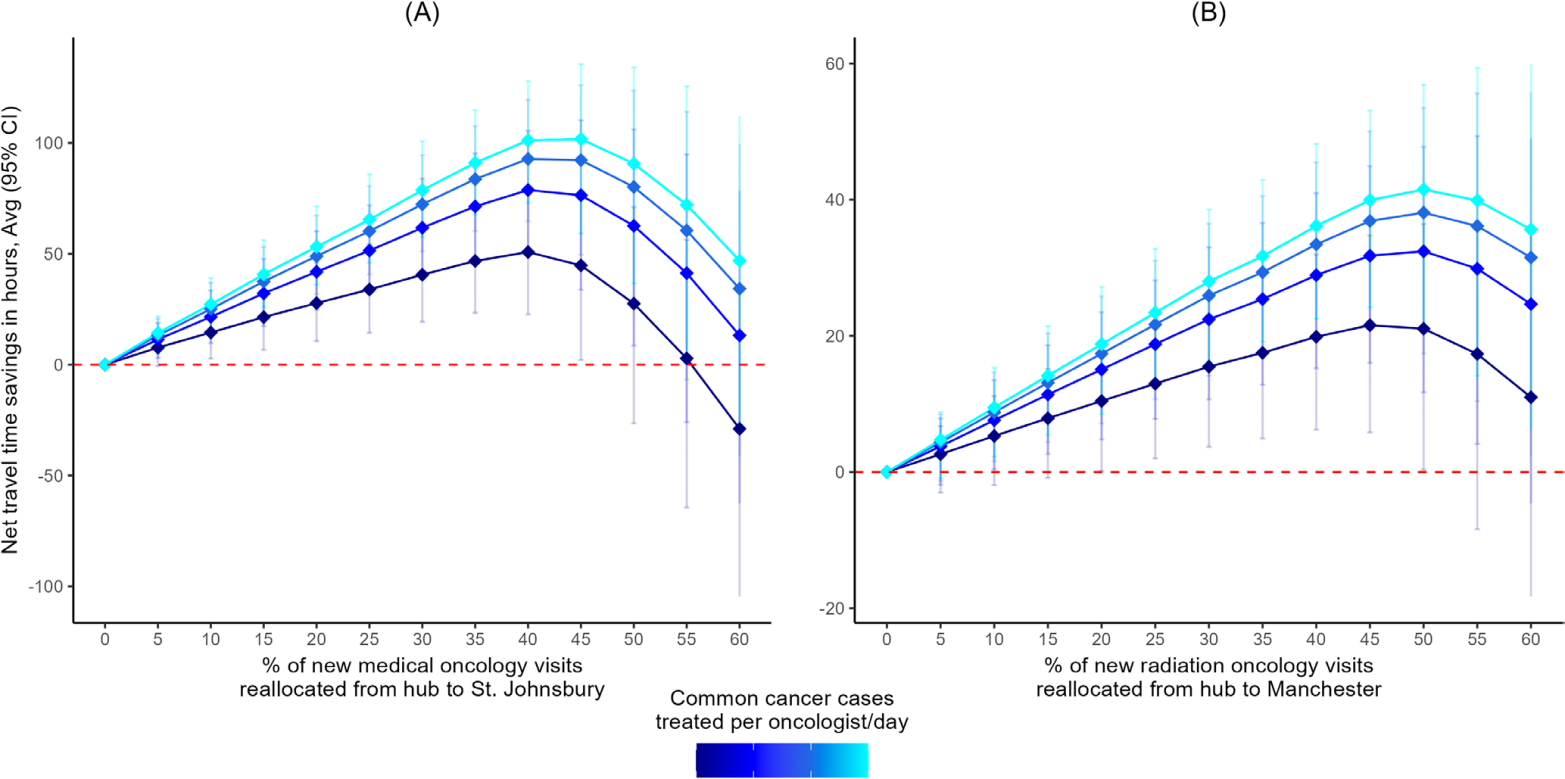
Net travel time savings as a function of oncology outreach. (A) In the most conservative case, at St. Johnsbury net savings were maximized by shifting 40% of new medical oncology visits, which resulted in 51 (95% CI: 23–71) total hours saved. For patients whose care was shifted to St. Johnsbury, this corresponded to 0.44 (95% CI: 0.20–0.67) hours saved per visit. (B) At Manchester, net savings were maximized by shifting 45% of new radiation oncology visits, which resulted in 22 (95% CI: 6–35) total hours saved. For patients whose care was shifted to Manchester, this corresponded to 0.49 (95% CI: 0.13–0.79) hours saved per visit.

We observed similar trends in the trajectory of net travel time savings for both St. Johnsbury and Manchester. As we flexed the number of patients with common cancers seen per day by an oncologist, net savings increased but at a diminishing rate (Figure 6).

In the most conservative case, for St. Johnsbury the round‐trip net savings significantly increased when shifting 5% to 45% of new medical oncology visits from the hub to satellite (Figure 6A). Net savings were maximized when reallocating 40% of new visits, resulting in 51 (95% CI: 23–71) total hours saved. For patients whose care was moved from the hub to the satellite, this corresponded to an average of 0.44 (95% CI: 0.20–0.67) hours saved per visit. When assuming follow‐up visits also occurred at the satellite, net savings increased to 609 (95% CI: 271–930) total hours (Table S2 & S3).

For Manchester, round‐trip net savings significantly increased when shifting 20% to 50% of new radiation oncology patients visits from the hub to satellite (Figure 6B). Net savings were maximized when reallocating 45% of new visits, resulting in 22 (95% CI: 6–35) total hours saved. For patients whose care was moved from the hub to the satellite, this corresponded to an average of 0.49 (95% CI: 0.13–0.79) hours saved per visit. When assuming follow‐up visits also occurred at the satellite, net savings increased to 194 (95% CI: 52–313) total hours (Table S2 & S4).

## 4 Discussion

In this study, we introduced a novel simulation framework for evaluating and optimizing oncology outreach policies. Using Dartmouth Health data for patients with common cancers, we found that St. Johnsbury and Manchester were viable sites for increased medical and radiation oncology outreach, respectively, and that optimizing outreach strategies at these satellites yielded over an hour in patient travel time savings per visit. If realized, such savings would not only enhance the patient experience (e.g., better travel time, financial burden, etc.), but could also result in improved clinical outcomes, health equity, and overall efficiency of the system.

The results from our test cases largely align with our hypotheses. We expected the trajectory of savings as a function of increased outreach to increase initially, reach a global maximum, and decline back towards (or below) zero. This is because increasing outreach reduces travel time for patients residing near a satellite [5], but reallocating too much oncologist effort can negatively impact those near the hub hospital. On the other hand, we were surprised that the optimized shifts fell within the 40% to 50% range, which suggest that reallocating about half of new medical and radiation oncology patient visits to St. Johnsbury and Manchester, respectively, is optimal for Dartmouth Health. These findings should be interpreted in the larger context of competing factors that need to be considered when evaluating outreach policies and do not necessarily reflect our recommendations for actual policy. For instance, our approach maximizes one unique element of patient access and does not consider the quality or cost of care. It is theorized that as one maximizes for patient access, the quality and/or cost of care diminishes [20]. Decision makers should carefully weigh these trade‐offs, as maximizing patient access may come at the expense of quality and/or cost. Additionally, our approach does not consider oncologist utility (e.g., financial benefit), preferences, or attrition due to lifestyle factors [12]. Considering these oncologist factors may provide a more comprehensive understanding of how much outreach is actually optimal.

Our approach is somewhat similar to existing methods that attempt to optimize physician outreach in outpatient settings but offers several new advancements. The study most closely related to ours proposed a method for optimizing physician outreach consultations within Veteran Affairs multi‐site care networks [11]. This was achieved by assigning physicians to patients in a manner that minimized the weighted sum of patient travel times and penalties for missed appointments (due to physician travel time). Our approach draws inspiration from this study and builds upon it in several ways. Similarly, we determined patient‐physician assignments by minimizing patient travel times; however, the trade‐off between patient and physician travel time was not modeled in the objective function but rather incorporated in our net travel time savings analysis. Second, we used a greedy algorithm for assigning patients to their closest available physician to respect the order of incident patients and streamline computational runtime. Electing to use a non‐greedy or globally optimal approach would fail to account for the chronological order of patients—resulting in unrealistic patient‐oncologist assignments—and introduce added computational overhead. Third, we estimated travel time savings by comparing counterfactual policy and control groups. This likely resulted in more conservative savings estimates compared to the previous approach, which quantified savings by comparing the optimized case to a baseline case that assumed all care was provided at centralized hospitals. Lastly, we relied upon stochastic simulations to generate distributions of travel time estimates, which allowed us to quantify uncertainty around savings estimates. In the savings plots, we observed that this uncertainty expanded with increasing levels of outreach (due to higher variation in the cost matrices), which is a major benefit in assessing the confidence of potential outreach policies.

So how exactly might our method be integrated into clinical practice? In its current form, our approach is well positioned to provide upstream decision support for oncology service line leads that determine staffing needs. We envision that our methods could be translated into a software solution that integrates within this staffing workflow and provides users with recommendations based on the latest data. Under the proposed model, administrative teams would pull de‐identified data from their health system's electronic medical record on a set cadence, input it into our software solution, and receive data/recommendations similar to what is presented in the current study. A future pilot within a health system is a critical next step to pressure test its utility.

While the presented use cases are specific to Dartmouth Health, our methods are not confined to a single health system or clinical service line. Health systems spanning larger geographic regions and those serving a predominantly rural catchment are increasingly motivated to implement outreach strategies, as they can yield higher margins by increasing patient volumes (revenues) at satellites and decreasing costs by moving care to less expensive outpatient settings [14]. Additionally, such methods can be extended to other disease areas where outreach strategies are common (e.g., cardiovascular disease, orthopedics, etc.) [21, 22], providing more economic opportunity. Furthermore, the paradigm shift from fee‐for‐service to value‐based care provides another financial incentive to adopt strategies that minimize patient travel burden [23]. Overall, we believe that health systems could gain valuable insight from integrating our methods into their staffing workflows; however, users should be wary of overreliance on one‐dimensional optimization (i.e., only considering patient travel time), recognizing that unaccounted factors may be critical for actual policy.

Our study has several limitations. First, our data was limited to patients with breast, colorectal, and lung cancers and only partially accounted for the case mix served within our catchment area. For instance, prostate cancer is another common cancer that can be clinically managed in community settings and thus benefit from outreach policies, but this data was not captured in our test cases. Second, as previously mentioned, we focused on maximizing one element of care delivery (patient access) and did not consider potential competing factors like oncologist preferences. We recognize that these unaccounted factors may be important but emphasize that our approach is nonetheless useful for thinking about directional investments in potential outreach strategies. Third, to reduce the complexity of patient‐physician assignments, we assumed that oncologists could provide care to patients of varying cancer types (i.e., no specialization). Even though > 80% of medical and radiation oncologists in our data treated multiple common cancers, this assumption may not fully align with the subspecialty model of care or provider preferences. Fourth, when calculating savings attributable to follow‐up visits, we assumed that patients repeatedly visited the same oncologist at the same site, regardless of the type of care they required. While simulating these assignments based on the type of care would be more realistic, incorporating these intricacies is highly challenging. Fifth, we did not incorporate a penalty for reduced clinic time that may be linked with physician travel burden. This is because Dartmouth Health oncologists typically commute to satellite clinics outside of business hours, but we recognize this may not generalize to other health systems. Sixth, the grid‐search approach for optimizing θ is computationally expensive. To produce the savings plots we opted for this approach, but in practice a heuristic approach would be preferable. Lastly, we have no empirical way of validating our results, as they are based on hypothetical simulations. A future pilot study would be greatly beneficial for evaluating our method's validity.

## 5 Conclusion

In this study, we introduced a novel simulation framework for evaluating and optimizing oncology outreach policies. Future directions include incorporating other elements of care delivery or stakeholder preferences into our simulations, extending our approach to optimize outreach strategies over a set of satellites (i.e., multisite optimization), and piloting an intervention study with a rural health system. Our approach is well positioned to provide upstream decision support for administrative leaders and can be leveraged to inform optimal resource allocations.

## Author Contributions

Conceptualization B.T.S., A.J.O., N.S.K., E.L.M; Methodology B.T.S., A.J.O., N.S.K., G.A.B., A.A.K., E.L.M; Software B.T.S; Data curation B.T.S., N.S.K., A.A.K., E.L.M; Investigation B.T.S., A.J.O., N.S.K., E.L.M; Validation B.T.S., E.L.M; Formal analysis B.T.S; Supervision B.T.S., E.L.M; Funding acquisition B.T.S., E.L.M; Visualization B.T.S; Project administration B.T.S., E.L.M; Resources B.T.S; Writing original draft B.T.S., A.J.O., N.S.K., G.A.B., E.L.M; Writing review and editing B.T.S., A.J.O., N.S.K., G.A.B., E.L.M.

## Funding

We would like to thank the National Cancer Institute at the National Institutes of Health for supporting this research (grant numbers R37CA263936 and T32CA260262), Dartmouth Health for supplying the data, and Research Computing at Dartmouth College for providing the computational resources for this work.

## Ethics Statement

Dartmouth College's institutional review board approved all study protocols and issued a waiver for obtaining informed consent from human subjects prior to commencement of the study.

## Conflicts of Interest

The authors declare no conflicts of interest.

## Supporting information


**Table S1:** Proportion of study cohort patients with at least one visit to a medical or radiation oncologist, stratified by cancer type and stage.
**Table S2:** Number of new patient visits that were reallocated in each test case.
**Table S3:** Travel time savings (in hours) and 95% confidence intervals attributable to shifting new medical oncology patient visits from Lebanon to St. Johnsbury.
**Table S4:** Travel time savings (in hours) and 95% confidence intervals attributable to shifting new radiation oncology patient visits from Lebanon to Manchester.

## Data Availability

The data that support the findings of this study are available on request from the corresponding author. The data are not publicly available due to privacy or ethical restrictions.
