# Supplementary material for: A Novel Simulation Framework for Evaluating and Optimizing Oncology Outreach Policies in a Regional Cancer System
Source: Cancer Med. 2026 Apr 5;15(4):e71771. doi: 10.1002/cam4.71771 (PMC13051838; doi:10.1002/cam4.71771)
Supplement: Supplementary file 1 — Table S1: Proportion of study cohort patients with at least one visit to a medical or radiation oncologist, stratified by cancer type and stage. Table S2: Number of new patient visits that were reallocated in each test case. Table S3: Travel time savings (in hours) and 95% confidence intervals attributable to shifting new medical oncology patient visits from Lebanon to St. Johnsbury. Table S4: Travel time savings (in hours) and 95% confidence intervals attributable to shifting new radiation oncology patient visits from Lebanon to Manchester. [file CAM4-15-e71771-s001.docx]

**Supplementary Methods. Details behind the simulator for a given set of inputs**

1. Sample incident patients and oncologist/site pairs
For a given set of inputs, a cost matrix is generated for the policy group, $C_{pol}$, and its counterfactual control, $C_{ctl}$. To form the row space of each matrix, a bootstrapped sample is obtained from the patient generator. Because each sampled patient’s cancer type and stage is known, it is determined if they require an oncologist (of the specified specialty) by drawing Bernoulli random variables from distributions parameterized by their respective cancer type and stage (Table S1).

The column space of each cost matrix varies across counterfactual groups. In the policy group, $\theta$ is used to determine how many new patient visits to reallocate to the satellite. Specifically, Lebanon-residing oncologists are uniformly sampled and reallocate up to 100% of their new patient visits to the satellite until the desired shift in visits is reached. This process results in a higher dimensional cost matrix for the policy group, with new oncologist/site columns (Figure 3). The order of columns in $C_{pol}$ is randomized and preserved in $C_{ctl}$.

Lastly, the elements of the cost matrices are populated with pairwise round-trip travel times between patients and oncologist/site pairs. Travel time is defined as the shortest path in minutes via road networks from the population-weighted centroid of a patient’s ZCTA to the corresponding site.

2. Systematically assign patients to oncologist/site pairs

Patients are assigned to oncologist/site pairs by the following logic:

For the $i$th patient and the $j$th oncologist/site let

$$C: Cost matrix of size m \mathrm{patients}\times n oncologists/sites, \mathrm{where} c_{ij}\in R^{+}$$

$$A:Assignment matrix of size m \mathrm{patients}\times n oncologists/sites, \mathrm{where} a_{ij}\in\left\{ 0,1 \right\}$$

$$k:Visit constraint vector for n oncologists/sites, \mathrm{where}k_{j}\in Z^{+}$$

then find

$$A^{*}=\arg\min_{A} \left\langle C,A \right\rangle=\arg\min_{A} \sum_{i=1}^{m} \sum_{j=1}^{n} c_{ij}a_{ij}$$

subject to

1. $\sum_{j=1}^{n} a_{ij}\leq1 \forall i\in\left\{ 1,\ldots, m \right\}$
2. $\sum_{i=1}^{m} a_{ij}\leq k_{j} \forall j\in\{1,\ldots, n\}$
3. $\sum_{j=1}^{n} a_{ij}\geq\sum_{j=1}^{n} a_{i+1,j}\forall i\in\left\{ 1,\ldots, m-1 \right\}$

In words, patients select the oncologist/site pair that minimizes their travel time with respect to the following constraints (corresponding to the roman numerals above):

1. Patients may only select one oncologist/site pair
2. Patients may only select an oncologist/site pair with available capacity
3. Patients select oncologist/site pairs on a first-come first-served basis

3. Calculate the total incurred patient travel time
After obtaining $A^{*}$, the total incurred patient travel time is calculated as the inner product between $C$ and $A^{*}$:

$$\left\langle C,A^{*} \right\rangle={\sum_{i=1}^{m} \sum_{j=1}^{n} c_{ij}a_{ij}^{*}=c}_{11}a_{11}^{*}+c_{12}a_{12}^{*} +\ldots+c_{mn}a_{mn}^{*}$$

The inner products for the policy and control groups are computed and stored in separate data structures before continuation to the next simulation iteration.

| **Table S1. Proportion of study cohort patients with at least one visit to a medical or radiation oncologist, stratified by cancer type and stage** | | | |
| --- | --- | --- | --- |
|  |  | Proportion of patients who saw at least one | |
| Cancer Type | Cancer Stage | Medical Oncologist | Radiation Oncologist |
| Breast | 1 | 0.34 | 0.15 |
|  | 2 | 0.39 | 0.25 |
|  | 3 | 0.43 | 0.24 |
|  | 4 | 0.54 | 0.11 |
| Colon and Rectum | 1 | 0.32 | 0.04 |
|  | 2 | 0.52 | 0.13 |
|  | 3 | 0.63 | 0.20 |
|  | 4 | 0.64 | 0.15 |
| Lung and Bronchus | 1 | 0.18 | 0.30 |
|  | 2 | 0.62 | 0.27 |
|  | 3 | 0.78 | 0.50 |
|  | 4 | 0.79 | 0.38 |

| **Table S2. Number of new patient visits that were reallocated in each test case** | | |
| --- | --- | --- |
|  | Number of unique patients whose care was moved | |
| Proportion of new patient visits shifted, $\theta$ | Lebanon to St. Johnsbury (medical oncology) | Lebanon to Manchester (radiation oncology) |
| 5% | 15 | 5 |
| 10% | 29 | 10 |
| 15% | 44 | 15 |
| 20% | 58 | 20 |
| 25% | 72 | 25 |
| 30% | 87 | 30 |
| 35% | 101 | 34 |
| 40% | 115 | 39 |
| 45% | 130 | 44 |
| 50% | 144 | 49 |
| 55% | 158 | 54 |
| 60% | 173 | 59 |
| 65% | 187 | 64 |
| 70% | 201 | 68 |
| 75% | 216 | 73 |
| 80% | 230 | 78 |
| 85% | 244 | 83 |
| 90% | 259 | 88 |
| 95% | 273 | 93 |
| 100% | 287 | 97 |

| **Table S3. Travel time savings (in hours) and 95% confidence intervals attributable to shifting new medical oncology patient visits from Lebanon to St. Johnsbury** | | | | |
| --- | --- | --- | --- | --- |
|  | Patient travel time savings | | Net travel time savings^a^ | |
| Proportion of new patient visits shifted, $\theta$ | New visits only | All visits^b^ | New visits only | All visits^b^ |
| 5% | 19 (10, 26) | 224 (124, 313) | 8 (-1, 15) | 92 (-7, 182) |
| 10% | 36 (24, 48) | 428 (286, 571) | 14 (3, 26) | 174 (32, 317) |
| 15% | 54 (39, 69) | 643 (465, 829) | 21 (7, 37) | 257 (80, 444) |
| 20% | 70 (53, 88) | 841 (636, 1062) | 28 (11, 46) | 333 (127, 553) |
| 25% | 87 (67, 107) | 1038 (803, 1283) | 34 (14, 54) | 407 (172, 652) |
| 30% | 104 (83, 126) | 1249 (994, 1515) | 41 (19, 63) | 487 (232, 752) |
| 35% | 121 (97, 144) | 1447 (1166, 1733) | 47 (23, 71) | 561 (281, 848) |
| 40% | 135 (107, 161) | 1618 (1280, 1938) | 51 (23, 77) | 609 (271, 930) |
| 45% | 140 (97, 174) | 1677 (1165, 2083) | 45 (2, 79) | 537 (25, 943) |
| 50% | 133 (79, 176) | 1593 (944, 2115) | 28 (-27, 71) | 330 (-318, 852) |
| 55% | 118 (51, 172) | 1419 (612, 2061) | 3 (-64, 56) | 34 (-773, 676) |
| 60% | 97 (22, 163) | 1169 (262, 1954) | -29 (-105, 36) | -347 (-1254, 437) |
| 65% | 74 (-8, 149) | 889 (-90, 1791) | -63 (-144, 13) | -751 (-1730, 152) |
| 70% | 48 (-38, 132) | 575 (-453, 1582) | -99 (-185, -15) | -1187 (-2215, -181) |
| 75% | 18 (-70, 107) | 218 (-842, 1279) | -140 (-228, -51) | -1675 (-2735, -615) |
| 80% | -10 (-101, 83) | -126 (-1207, 998) | -179 (-269, -85) | -2142 (-3223, -1019) |
| 85% | -39 (-130, 58) | -470 (-1564, 691) | -217 (-309, -121) | -2609 (-3703, -1448) |
| 90% | -71 (-162, 27) | -846 (-1940, 329) | -260 (-351, -162) | -3117 (-4210, -1942) |
| 95% | -100 (-190, -1) | -1197 (-2282, -7) | -299 (-390, -200) | -3591 (-4676, -2400) |
| 100% | -130 (-223, -30) | -1558 (-2676, -364) | -339 (-433, -240) | -4074 (-5192, -2880) |
| ^a^Statistics reported for the most conservative case, where oncologists provide care to an average of 4 patients with breast, colorectal, or lung cancer per day  ^b^In our data, patients had an average of 12 medical oncology visits | | | | |

| **Table S4. Travel time savings (in hours) and 95% confidence intervals attributable to shifting new radiation oncology patient visits from Lebanon to Manchester** | | | | |
| --- | --- | --- | --- | --- |
|  | Patient travel time savings | | Net travel time savings^a^ | |
| Proportion of new patient visits shifted, $\theta$ | New visits only | All visits^b^ | New visits only | All visits^c^ |
| 5% | 6 (0, 10) | 55 (4, 92) | 3 (-3, 7) | 24 (-27, 61) |
| 10% | 12 (5, 18) | 110 (45, 163) | 5 (-2, 11) | 48 (-17, 101) |
| 15% | 18 (10, 26) | 165 (86, 230) | 8 (-1, 15) | 71 (-8, 136) |
| 20% | 24 (14, 33) | 219 (127, 295) | 10 (0, 19) | 94 (1, 170) |
| 25% | 30 (19, 40) | 273 (175, 358) | 13 (2, 22) | 117 (18, 201) |
| 30% | 36 (25, 47) | 327 (221, 422) | 15 (4, 26) | 139 (33, 234) |
| 35% | 41 (29, 52) | 371 (257, 471) | 18 (5, 29) | 158 (44, 258) |
| 40% | 47 (33, 59) | 423 (300, 532) | 20 (6, 32) | 179 (56, 287) |
| 45% | 52 (36, 65) | 470 (328, 588) | 22 (6, 35) | 194 (52, 313) |
| 50% | 55 (34, 71) | 496 (310, 635) | 21 (0, 36) | 189 (3, 328) |
| 55% | 55 (29, 74) | 494 (263, 670) | 17 (-8, 37) | 156 (-76, 331) |
| 60% | 52 (23, 76) | 468 (205, 687) | 11 (-18, 35) | 99 (-164, 318) |
| 65% | 48 (18, 76) | 430 (158, 680) | 3 (-27, 31) | 29 (-243, 279) |
| 70% | 44 (12, 74) | 394 (109, 663) | -4 (-35, 26) | -32 (-317, 237) |
| 75% | 38 (6, 70) | 345 (50, 634) | -12 (-45, 20) | -112 (-407, 177) |
| 80% | 33 (-1, 66) | 295 (-7, 591) | -21 (-55, 11) | -193 (-496, 103) |
| 85% | 27 (-7, 61) | 244 (-62, 549) | -31 (-65, 3) | -276 (-582, 29) |
| 90% | 21 (-13, 56) | 192 (-119, 508) | -40 (-74, -5) | -359 (-670, -43) |
| 95% | 15 (-20, 51) | 139 (-178, 459) | -49 (-85, -14) | -444 (-761, -124) |
| 100% | 11 (-26, 47) | 95 (-230, 421) | -57 (-93, -21) | -513 (-838, -187) |
| ^a^Statistics reported for the most conservative case, where oncologists provide care to an average of 4 patients with breast, colorectal, or lung cancer per day  ^b^In our data, patients had an average of 9 radiation oncology visits | | | | |
